# Is preexisting immunity to seasonal coronaviruses limited to cross-reactivity with SARS-CoV-2? A seroprevalence cross-sectional study in north-eastern France

**DOI:** 10.1016/j.ebiom.2021.103580

**Published:** 2021-09-12

**Authors:** Hadeel T. Zedan, Gheyath K. Nasrallah

**Affiliations:** a Department of Biomedical Science, College of Health Sciences, Member of QU Health, Qatar University, P.O. Box 2713, Doha, Qatar; b Biomedical Research Center, Qatar University, Doha, Qatar

In light of the ongoing coronavirus disease 2019 (COVID-19) pandemic caused by the severe acute respiratory syndrome coronavirus 2 (SARS-CoV-2), there remain unanswered questions regarding the humoral immune response and the breadth of cross-reactivity with preexisting seasonal human coronaviruses (HCoVs). Initially, the lack of preexisting immunity was believed to contribute to the virus's rampant spread. Several studies reported that the clinical manifestations of COVID-19 are age-dependent, with children appearing to be less susceptible to infection and severe disease [1]. This highlighted the potential cross-protective effect of preexisting immunity to seasonal HCoVs. However, reported data on cross-reactivity was contradictory, antigen-dependent, and mostly conducted on small sample sizes.

Among all HCoVs, four globally circulating seasonal strains are known to cause mild common colds, whereas three cause severe infections, including the currently circulating SARS-CoV-2. Seasonal HCoVs (HCoV-HKU1, -OC43, -229E, and -NL63) have remarkable similarities with the envelope, membrane, and nucleocapsid protein sequence of SARS-CoV-2; 96%, 91%, and 91%, respectively, whereas they only share about 24-30% of the spike (S) protein sequence [2]. Importantly, due to the pronounced similarity between these HCoVs, cross-reactivity between the cognate antibodies targeting SARS-CoV-2 structural proteins can be reasonably expected [2].

To characterize the humoral immunity to SARS-CoV-2 and seasonal HCoVs, Woudenberg et al. measured the antibody responses to different antigens using three different assays in a large cohort of children (≤10 years), adults (11-50 years), and elderly (>50 years) admitted to the hospital for non-COVID-19 [3]. A total of 2545 samples, including 90 pre-pandemic samples, collected from 11 hospitals in north-eastern France were included. As described in the earlier issue of EBioMedicine, this cross-sectional study reported an age-related immune response to SARS-CoV-2 with a seroprevalence ranging between 7-8%. Precisely, young adults exhibited lower antibody responses compared to children and elderly. These findings (summarized in Fig. 1) are in line with previous data reported in SARS and COVID-19 patients, where advanced age correlated with early responses and higher antibody titers, possibly due to the priming effect from preexisting HCoVs [4, 5].

**Fig. 1:**
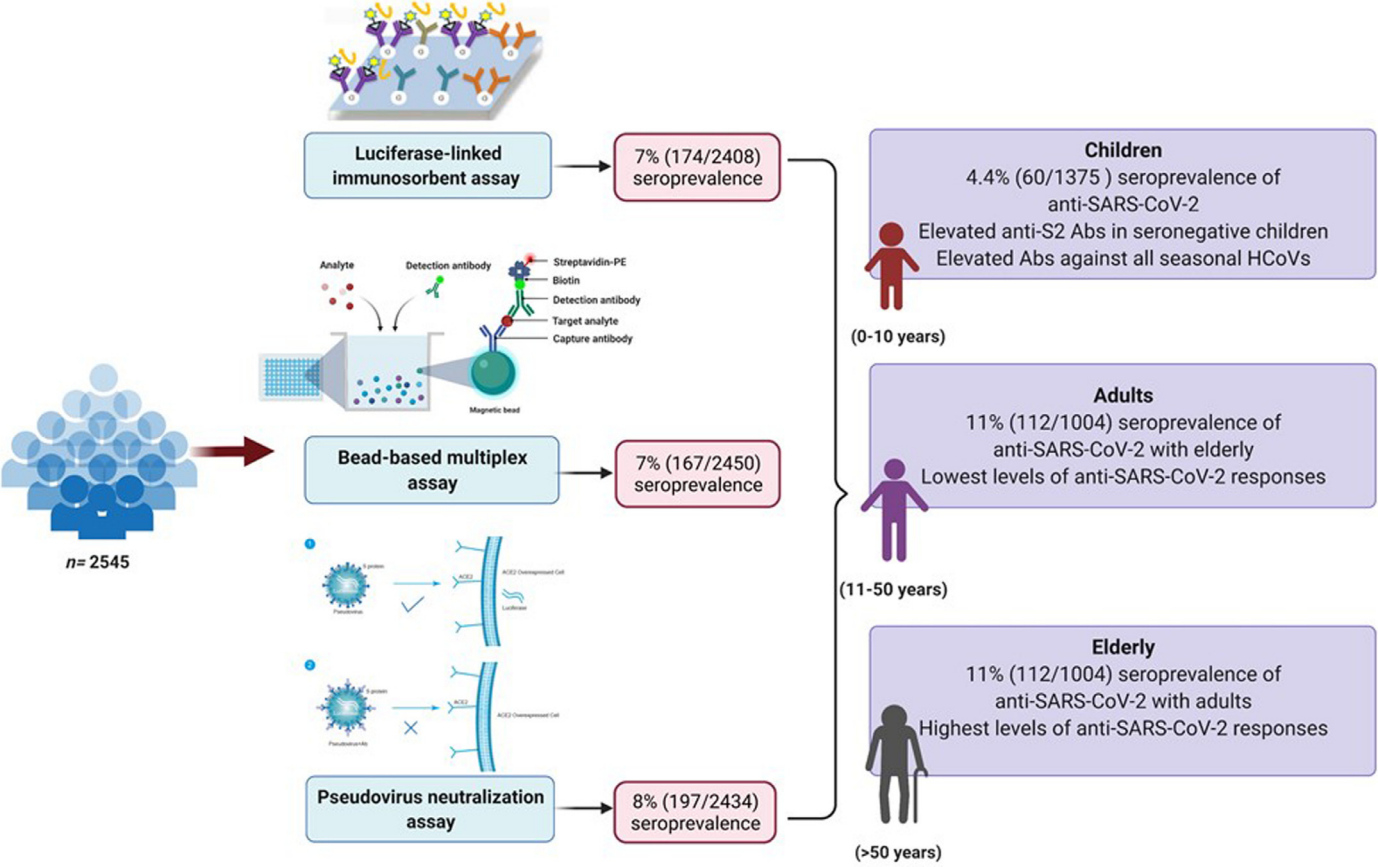
A Graphical abstract highlighting the main findings of Woudenberg et al. study. The figure was Created with BioRender.com.

All SARS-CoV-2 seropositive individuals had high levels of antibodies to all seasonal HCoVs, which substantially increased with age (up to 10 years), particularly to HCoV-229E. Strong evidence of cross-reactivity with HCoV-OC43 was also observed across the whole age spectrum, where higher anti-S IgG were positively correlated with anti-SARS-CoV-2 responses. This could reflect a higher affinity of SARS-CoV-2 antibodies to HCoV-OC43 S-protein. In fact, elevated levels of preexisting anti-HCoV-OC43 IgG were also reported in SARS patients, showing more than 4-fold increase in paired acute/convalescent samples [6]. Further, IgG titers in convalescent plasma of COVID-19 patients were higher against HCoV-OC43 S-protein than HCoV-229E, despite the presence of reactive memory B-cells to both viruses in seronegative subjects [7].

Notably, elevated antibody level against the S2-subunit of SARS-CoV-2 was observed among seronegative children. Considering that the S2-subunit exhibits a higher degree of homology than S1, it is more likely to be the main target of cross-reactive antibodies [8]. This could be explained by the qualitative differences in the antibody repertoires between children and adults, which reflect higher exposure of children to seasonal HCoVs compared to adults.

Woudenberg et al. reported that among SARS-CoV-2 seronegative individuals, there was significant cross-reactivity between anti-S antibodies targeting seasonal HCoVs and SARS-CoV-2. However, no significant evidence of cross-protective effect from SARS-CoV-2 infection was observed. Hence, the authors concluded that preexisting immunity against seasonal HCoVs is more likely to be associated with cross-reactivity between assays, but not necessarily leads to cross-protection. The strength of this study comes from involving a large cohort and extensively characterizing the cross-reactive humoral immunity by covering all SARS-CoV-2 structural proteins and seasonal HCoVs S-protein. Accordingly, this study provided empirical evidence of the high propensity for antibody cross-reactivity, which is critical information for understanding immunity to coronaviruses.

The implications of Woudenberg et al. study are two-fold; first, preexisting immunity against seasonal coronaviruses is more likely to be associated with cross-reactivity rather than cross-protection. Second, the antibody response is lower in young adults compared to children and elderly, particularly anti-S2 antibodies. However, it should be noted that cellular immunity also plays an important role as preexisting T-cell immunity to seasonal HCoVs can prime the response to SARS-CoV-2 [4]. Therefore, together with preexisting B-cell and T-cell memory, antibody cross-reactivity between SARS-CoV-2 and seasonal HCoVs may have significant ramifications on natural infection. Based on the knowledge obtained from epidemiological studies on HCoVs, cross-protective immunity following prior or repeated infections by seasonal HCoVs is improbable to be sterilizing or long-lasting [8]. However, it could reduce transmission or disease severity. Regardless, it is imperative that any effect, positive or negative, of preexisting HCoV-elicited immunity on the natural course of SARS-CoV-2 infection to be fully delineated. Also, it is of paramount importance to understand to what extent, if any, the humoral immune response to SARS-CoV-2 can contribute to virus-induced immunopathogenesis.

## Contributors

Conceptualization, data curation, investigation, visualization, writing-original draft: H.T.Z.; validation, writing-review and editing: H.T.Z. and G.K.N.; funding acquisition: G.K.N.

## Declaration of Competing Interest

The authors declare no conflict of interest.
